# Healthcare utilisation among patients with stress-induced exhaustion disorder treated with a multimodal rehabilitation programme – a longitudinal observational study

**DOI:** 10.1186/s12888-022-04300-8

**Published:** 2022-10-13

**Authors:** Fredrik Norström, Lisbeth Slunga Järvholm, Therese Eskilsson

**Affiliations:** 1 Department of Epidemiology and Global Health, Umeå University, SE-901 87 Umeå, Sweden; 2 Department of Public Health and Clinical Medicine, Section of Sustainable Health, Umeå University, Umeå, Sweden; 3 Department of Community Medicine and Rehabilitation, Section of Physiotherapy, Umeå University, Umeå, Sweden

**Keywords:** Health-related quality of life, Healthcare consumption, Clinical burnout, Northern Sweden

## Abstract

**Background:**

Stress-induced exhaustion disorder is a major challenge in Swedish working life. Despite its increase in prevalence, there is still limited knowledge about the effectiveness of different rehabilitation methods. In this study, we aim to describe the healthcare utilisation for patients with stress-induced exhaustion disorder before, during and after a multi-modal rehabilitation (MMR) programme, as well as the health-related quality of life, work ability, sick leave level and psychological measures, and their possible relations.

**Methods:**

In this longitudinal observational study, 53 patients who were part of an MMR programme at the Stress Rehabilitation Clinic participated with survey data, and among them 43 also contributed with healthcare data. Data were collected from one year before start of MMR to one year after the end of it. The patients also answered a questionnaire at the start of, end of and at a one-year follow-up of the MMR, which included questions about health-related quality of life, work ability, clinical burnout, sick leave level, anxiety and depression.

**Results:**

There was a statistically significant increase in healthcare consumption during MMR, if including visits to the Stress Rehabilitation Clinic, while it decreased if excluding such visits, when comparing with before and after MMR. During the follow-up period there was a non-statistically significant (*p*=0.11), but still rather large difference (15.4 compared with 12.0 visits per patient), in healthcare consumption in comparison with the period before MMR, when excluding follow-up visits at the Stress Rehabilitation Clinic. Health-related quality of life was rated as poor before MMR (mean 0.59). There was a statistically significant improvement, but values were still below normal at the end of follow-up (mean 0.70). In addition, the level of sick leave, the work ability and signs of clinical burnout improved statistically significantly after MMR, but were not fully normalised at the end of follow-up. Individual healthcare consumption was related to residual health problems.

**Conclusions:**

Patients with stress-induced exhaustion disorder have not reduced their healthcare consumption notably after MMR, and residual health problems remain for some patients. More studies are needed for a deeper understanding of the individual effectiveness of MMR, and also of its cost-effectiveness.

## Background

Psychiatric illnesses are the most common reason for long-term (at least 60 days) sick leave in Swedish working life [1]. Their proportion of total sick leave has increased over time, accounting for almost half of the sick leave cases among women and over a third of the cases for men during 2017–2019. A large part of the psychiatric diseases that caused sick leave periods of more than 14 days consisted of common mental disorders and especially stress-related disorders.

Prolonged exposure to stress can lead to burnout, but there is no general agreement on a clinical diagnosis of burnout. In Sweden, the new diagnosis stress-induced exhaustion disorder is classified as diagnosis F43.8A according to the 10th revision of the International Statistical Classification of Diseases and Related Health Problems (ICD-10) and was established in 2005 by the National Board of Health and Welfare. It has since been used in Swedish healthcare and can be considered a valid clinical equivalent to burnout [2]. Internationally other equivalents are used for this condition [3]. Typical of the condition is a markedly reduced mental energy, lack of endurance, and a prolonged recovery time after mental effort. Core symptoms are cognitive impairments and sleep disturbances [2], often in combination with somatic [4] and mental symptoms [5], that may remain as long as seven years after seeking initial care in severe cases [6].

For patients suffering from stress-induced exhaustion disorder, and similar equivalent disorders, it is often a challenge to recover and return to work. The employee needs support to improve his/her chances of going back to even part time work. Different interventions have tried to increase the chances for these patients to improve their health as well as regaining working ability. Examples of interventions are cognitive behavioural therapy [7–12], physical activities [11, 13], and workplace-oriented interventions [14–16], which have also commonly been combined with multimodal rehabilitation (MMR) programmes that have been developed and investigated jointly. Most of the studies have focused on the outcomes of returning to work and psychological measures such as burnout, anxiety and depression [9, 12, 17].

Stress-induced exhaustion disorder has increased a lot in Sweden for both men and women over the last 15 years [1]. The costs for sick leave due to psychiatric diseases are high. The insurance company Skandia estimated the costs from sick leaves in Sweden to be 64 billion Swedish krona (6.9 billion US dollars on 18 February, 2022) [18]. However, the costs for stress-induced exhaustion disorder has not been presented by itself. It is rare with studies investigating healthcare utilisation, and how it changes for the patient over time [19], and complementary information are therefore of importance as support in cost estimates for the disease. It is also rare with studies on health-related quality of life for the patients [12]. Our study contributes with information about cost and health-related quality of life that are needed for a cost-utility analysis of interventions for stress-induced exhaustion disorder such as MMR, which is needed in the prioritization of resources within the healthcare.

In the current study, an MMR programme was used for patients on sick leave due to stress-induced exhaustion disorder that were referred to a specialised Stress Rehabilitation Clinic in a municipality in Northern Sweden. The main aim of this study was to describe the healthcare utilisation for patients with stress-induced exhaustion disorder before, during and after an MMR programme. Secondary aims were to study health-related quality of life, the patients’ estimated work ability, the level of sick leave and psychological measures during the same period and their possible relations to the amount of healthcare utilisation. We hypothesised that healthcare utilisation due to stress-induced exhaustion disorder and related diagnoses would be notably less after going through an MMR programme.

## Methods

### Study design and participants

In this longitudinal observational study, patients who were part of an MMR programme at the Stress Rehabilitation Clinic at the University Hospital in Umeå in Sweden were recruited from March to October 2015. This clinic specialises in the treatment of stress-induced exhaustion disorder and receives referrals mainly from primary healthcare. In the current study, patients who had a diagnosis of stress-induced exhaustion disorder (ICD-10 code F43.8A) were invited to participate. Further inclusion criteria were: 18–60 years of age, a sick leave of at least 50%, employment, and considered suitable for group-based stress rehabilitation where the exchange of experiences between the participants is an important component. Patients were not invited if they had already participated in another intervention study, had another disease that worsened the chances of return to work, had disability pension, had activity or sickness compensation, or were self-employed.

**Fig. 1:**
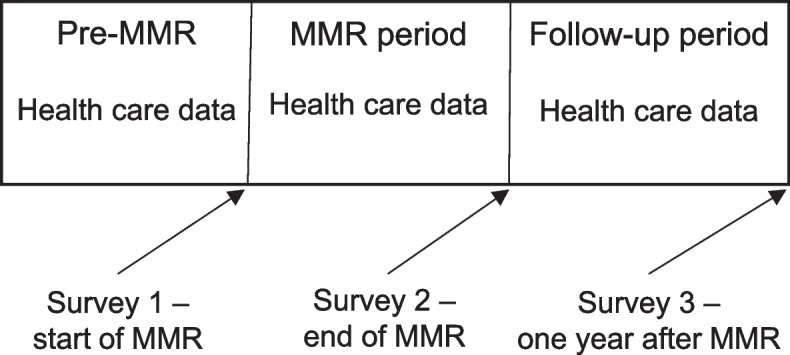
Illustration of study periods

The study period ranged from the year before the MMR programme until one year after the end of the MMR programme from which we defined three periods to be used in analyses; pre-MMR period, MMR period and follow-up period. The participants filled in three surveys, the first one at the start of the MMR programme, i.e. baseline, the second at the end of the MMR programme and the third around a year after the MMR programme. At baseline, information about age, sex, occupation and education was collected. All the surveys collected information about participants’ work ability, level of burnout, anxiety, depression, and health-related quality of life. Participants were also asked about permission to collect data about their healthcare utilisation for the whole study period from the Region Västerbotten’s register. The period for the study is illustrated in Fig. 1.

In total, 271 patients were diagnosed with stress-induced exhaustion during the recruitment period and of them 82 fulfilled the criteria for the current study. Of these 82 patients, 53 individuals agreed to participate in our study and completed MMR (29 patients declined to participate in the full MMR) and 51 of them responded to all the surveys during the study time. Furthermore, there were data about healthcare utilisation for 43 of the 53 participants, collected from the Region Västerbotten’s register after informed consent for it had been received from them. The Regional Ethical Board in Umeå, Sweden, approved the study (Dnr 2015/49-31Ö, Dnr 2017/524-32).

### The multimodal rehabilitation

All participants took part in a 24-week MMR programme. The MMR programme consisted of 22 weekly three-hour group sessions based on cognitive behavioural therapy, the aim of which was to support behavioural change concerning the balance between activation and recuperation [20]. Each session started with relaxation, followed by specific themes with the aim of providing knowledge and strategies for healthy behaviour, amongst them how to deal with stress and emotions, strategies for sleep, recovery and physical activity. The cognitive behavioural therapy also included two individual meetings with the group leader (licensed psychologist/psychotherapist), to discuss and evaluate concrete activities based on the individual's values [9]. The MMR programme also included a dialogue-based workplace intervention with a convergence meeting where the manager and the employee were guided by a healthcare rehabilitation coordinator (in this study a specially trained physiotherapist) to find concrete solutions to enable return to work [21, 22]. The content of the group meetings and the structure of the dialogue-based workplace intervention were standardized, while the individual activities to achieve healthy behaviour and return to work could vary somewhat. Regular follow-up visits were made to a physician during the MMR.

### Healthcare utilisation data

The healthcare utilisation data comprised only outpatient care in the region’s public healthcare and we only included ICD-10 diagnoses that we considered related to the stress-induced exhaustion disorder. In addition to mental and behavioural disorders, we therefore also studied healthcare use connected to diseases of the circulatory system, the musculoskeletal system and symptomatic diagnoses. In Table 1, the blocks of diseases according to the ICD-10 system that were studied are listed.

**Table 1:** Number of patients with registered ICD-10 diagnoses belonging to diagnostic blocks that were assessed as possibly related to stress-induced exhaustion disorder among the 43 study participants. Stress-induced exhaustion disorder (F43.8A) is included in these data

Diagnosis	Pre-MMR		MMR period		Follow-up period		During study period	
	n	%	n	%	n	%	n	%
Mental and behavioral disorders (F00–F99)	35	81%	43	100%	29	67%	43	100%
Diseases of the circulatory system (I00-I99)	5	12%	4	9.3%	3	7%	6	14%
Diseases of the musculoskeletal system and connective tissue (M00-M99)	6	14%	7	16%	5	12%	14	33%
Symptoms, signs and abnormal clinical and laboratory findings, not elsewhere classified (R00-R99)	11	26%	6	14%	5	12%	18	42%

*MMR* Multimodal rehabilitation, *ICD-10* International classification of diseases, tenth revision

For each visit/contact, the data retrieved from the region’s register included information about diagnoses registered, contact form, e.g. physical or telephone/telemedicine meeting, and the occupational category of the personnel at the visit, e.g. nurse or physiotherapist. The patient contacts included in our study consisted of visits to primary care centres, specialist clinics in hospitals and public occupational healthcare, which are available to employees in the county council, and telemedicine visits, while for instance telephone contacts were excluded. In the county of Västerbotten the majority of primary care contacts are made in public healthcare, but occupational healthcare is to a large extent located in the private sector. The average number of healthcare contacts is reported, and results are presented separately for the three periods of the study. Data are also presented with and without healthcare contacts at the Stress Rehabilitation Clinic.

### Questionnaires

Six of the questions from the Short Form 36 item questionnaire (SF-36) [23], usually referred to as SF-6, were used to measure health-related quality of life. The questions from SF-6 were translated to an index ranging from 0 to 1, with 0 corresponding to death and 1 to full health [24].

The level of sick leave for participants was decided based on questions about their labour market activity in percentage and their current level of sick leave in %. There were five levels of sick leave to choose between: 0%, 25%, 50%, 75% and 100% of sick leave.

Work ability was measured with the Work Ability Index (WAI). WAI consists of questions related to seven different dimensions and have already been used in many studies [25]. For each of these dimensions the responses are translated to a score of 1–7 and an index can then be created by adding these scores. This index is recommended to be grouped by the scores 7–27, 28–36, 37–43 and 44–49 to represent level of work ability, ranging from poor to excellent.

The Shirom Melamed Burnout Questionnaire (SMBQ) was used to measure the level of burnout. The SMBQ consists of 22 questions with seven severity levels and has been validated for the Swedish setting [26]. We coded the responses in level of severity as 1–7 and combined these with an average level of ≥ 4.4 defining exhaustion, which is in line with previous studies [26].

The Hospital Anxiety and Depression (HAD) scale, constructed by Zigmond and Snaith and widely used [27], was used to measure anxiety and depression. HAD consists of 14 questions, which are usually separated into seven questions each for HAD anxiety and HAD depression. Each question has four response alternatives, which were coded as 0-–3 and thereafter added together to a score ranging from 0 to 21. A score up to 7 is considered normal, 8–10 mild problems, and 11 and higher severe problems [27].

### Statistics

Descriptive statistics were presented using frequency tables, cross-tabulations, and mean and median values. Means were compared using Student’s t-test. Paired t-test was used to make comparisons between time points. Spearman’s rank correlation was used for associations between survey data and healthcare utilisation. Considering the small sample, correlations, including the *p*-values from the test, should be seen as explorative analyses and be interpreted carefully. Also other analyses should be interpreted cautiously. In some of our analyses, results were presented both with and without visits at stress the rehabilitation clinic, while for other analyses we only presented with visits at the stress rehabilitation clinic. Statistical significance was defined at the 5% level. Stata 13.1 (Stata-Corp LP, College Station, TX) was used for statistical analysis and Microsoft Access for data handling.

## Results

Of the 51 participants who responded to all surveys, there were 41 (80%) women, 26 (51%) who had a university degree, and they were 29 to 60 years old with a median age of 43 years and a mean age of 43.1 years (standard deviation of 8.1 years). Around half (*n* = 25) of the participants worked in the private sector, and the rest in the public sector (*n* = 12 in municipalities, *n* = 11 in county councils and *n* = 3 in governmental work places). Among the eight patients that did not agree to grant access to their healthcare utilisation data, there was only one man, one person with a university degree, and they were also more commonly working in the private sector (*n* = 5), while the mean age was similar to the whole population.

### Results for healthcare utilisation

**Fig. 2:**
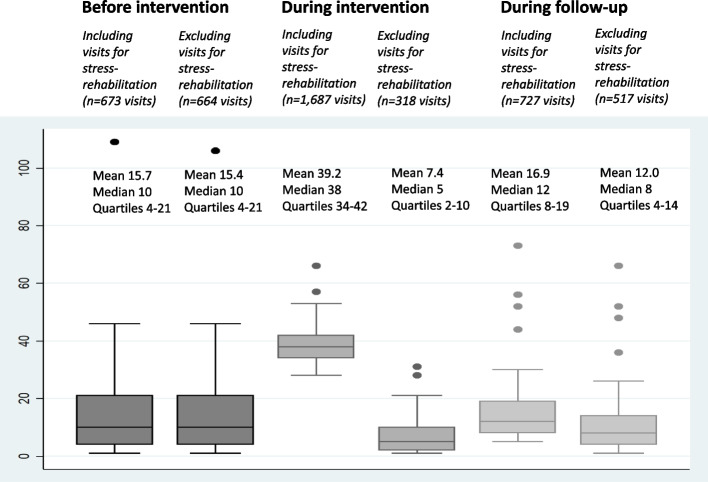
Healthcare visits during study

Block F (F00-F99), which involves mental and behavioural disorders, was the most common reason for using healthcare in the 43 patients during the whole study period. Healthcare was to some degree also given for diagnoses in the other three blocks of diseases (Table 1). Taken all together, the 43 patients had 3087 visits, with a median of 65 visits (quartiles 51 and 80) and a mean of 72.0 visits (standard deviation 35.0), to different healthcare facilitators during the whole study period (Fig. 2). The majority of the visits were during the MMR programme period, where there were statistically significantly more visits than during other time periods. These visits were mainly made at the Stress Rehabilitation Clinic with only 19% (*n* = 318) of the visits elsewhere. Considering only visits outside the Stress Rehabilitation Clinic, there were even statistically significantly fewer visits during MMR, with an average of 7.4 visits, compared to other time points. However, this difference between pre-MMR and the follow-up period was not statistically significant (*p* = 0.11), despite a rather large difference (15.4 compared with 12.0 visits per patient) between the time periods.

Only a few of the visits elsewhere were made at the occupational healthcare for people employed at the county council (*n* = 27) or consisted of a telemedicine visit (*n* = 3). Among other visits, 976 (32%) were to primary care and 2081 (68%) were to specialist clinics. Of the 3087 visits, there were 1912 individual meetings (62%), 1148 group meetings with two or more patients and healthcare personnel present, and 27 meetings had no such information. Outside the Stress Rehabilitation Clinic only 92 visits were not individual meetings, thus group meetings were mainly made at the Stress Rehabilitation Clinic.

Participants’ visits to physiotherapists and psychologists occurred mainly during the MMR intervention (Table 2). Before and after the MMR programme visits to physicians were on similar levels, while visits to physicians during the MMR period were 50% more frequent when data from the Stress Rehabilitation Clinic were included. Considering only meetings outside this clinic, the meetings with physicians were instead considerably fewer (107 compared with 208 and 188 during the other periods).

**Table 2:** Occupations visited during the study period^a^

	Pre-MMR		MMR period		Follow-up period		During study	
	Including stress-rehabilitation (*n*=695)	Excluding stress- rehabilitation (*n*=686)	Including stress-rehabilitation (*n*=1,799)	Excluding stress- rehabilitation (*n*=328)	Including stress-rehabilitation (*n*=801)	Excluding stress- rehabilitation (*n*=577)	Including stress-rehabilitation (*n*=3,259)	Excluding stress- rehabilitation (*n*=1,591)
Physician	213	208	300	107	208	188	721	503
Nurse	85	85	48	48	77	77	210	210
Physioterapeut	119	118	592	63	178	99	889	280
Occupational terapeut	7	7	6	6	31	31	44	44
Welfare officer	72	72	28	28	37	37	137	137
Psychologist	86	83	752	3	197	72	1,035	158
Other occupation	113	113	73	73	73	73	259	259

^a^During a meeting, one or more occupations was met, on a few occasions even two or more from same group above

*MMR* Multimodal rehabilitation

### Results from questionnaires

Participants rated their health-related quality of life on average low before MMR with a mean value of 0.59 (Table 3). There was a statistically significant improvement between time points, but the health-related quality of life remained below the normal range during the study period (mean value of 0.70 at end of follow-up).

**Table 3:** Results from questionnaires for the 51 participants

		Pre-MMR	MMR period	Follow-up period
Short form 36 – index value		*n*=51	*n*=51	*n*=40
	Mean (SD)	0.59 (0.049)	0.68 (0.099)	0.70 (0.108)
	Median (1^st^-3^rd^)^a^	0.59 (0.56-0.62)	0.66 (0.62-0.70)	0.70 (0.64-0.78)
	p^b^	-	<0.001	0.048
Sick leave		*n*=51	*n*=51	*n*=51
	0%	0	3 (6%)	32 (63%)
	25%	0	13 (25%)	7 (14%)
	50%	11 (22%)	12 (24%)	8 (16%)
	75%	6 (12%)	9 (18%)	0
	100%	34 (67%)	14 (27%)	4 (7.8%)
	p^b^	-	<0.001	<0.001
Work ability		*n*=51	*n*=47	*n*=47
	Poor	43 (84%)	18 (38%)	13 (28%)
	Moderate	8 (16%)	23 (49%)	18 (38%)
	Good	0	6 (13%)	16 (34%)
	Excellent	0	0	0
	Mean (SD)	22.1 (5.1)	28.2 (6.1)	32.4 (7.6)
	Median (1^st^-3^rd^)	21.5 (18-26)	28 (25-31)	35 (27-38)
	p^b^	-	<0.001	<0.001
Shirom Melamed Burnout Questionnaire		*n*=51	*n*=50	*n*=48
	Participants with ≥4.4	49 (96%)	19 (36%)	9 (19%)
	Mean (SD)	5.53 (0.66)	3.84 (1.15)	3.55 (1.13)
	Median (1^st^-3^rd^)	5.7 (5.0-6.0)	4.0 (2.9-4.6)	3.5 (3.0-4.2)
	p^b^	-	<0.001	0.011
The Hospital Anxiety and Depression scale	*Anxiety*	*n*=51	*n*=50	*n*=48
	≥11	25 (49%)	5 (10.0%)	3 (4.2%)
	Mean (SD)	10.0 (3.2)	6.2 (3.4)	5.6 (3.5)
	Median (1^st^-3^rd^)	10 (8-12)	6 (4-8)	5 (3-7.5)
	p^b^	-	<0.001	0.097
The Hospital Anxiety and Depression scale	*Depression*	*n*=51	*n*=50	*n*=48
	≥11	16 (31%)	2 (4.0%)	1 (2.1%)
	Mean (SD)	8.7 (3.0)	4.4 (2.9)	3.8 (2.8)
	Median (1^st^-3^rd^)	9 (7-11)	4 (2-6)	3.5 (1-5.5)
	p^b^	-	<0.001	0.079

*MMR* Multimodal rehabilitation, *SD* Standard deviation

^a^1^st^ and 3^rd^ quartiles

^b^Comparing with previous measurement time with a paired t-test

At baseline, 34 (67%) of the participants were on full-time sick leave, while six (12%) worked 25% and 11 (22%) halftime (Table 3). The level of sick leave among participants reduced during the study time; nevertheless, at the end of the study, 37% (*n* = 19) of the participants were on sick leave to some degree, although only four (7.8%) had more than 50% sick leave. There was a statistically significant reduction of sick leave level by time.

The participants improved their work ability after MMR and during the 1-year follow-up, but even so most of the participants still had a reduced work ability, with 66% still showing poor or moderate work ability (Table 3).

Only two of the 51 participants who responded to the SMBQ had a value below 4.4 at baseline, which was used as a threshold level for significant exhaustion. Most of the participants recovered during the study time to an SMBQ value below this after the MMR programme, though 19% (*n* = 9) still had signs of burnout/exhaustion at the 1-year follow-up, (Table 3).

At baseline, almost half (*n* = 25) of the participants had severe problems with anxiety and 16 (31%) had problems with depression (Table 3). At the end of the MMR programme, only two participants had severe problems with depression and five with anxiety. The further improvement from post MMR to the 1-year follow-up was not statistically significant although numerically a tendency towards a better situation was found.

### Association between healthcare utilisation and survey variables

There was a statistically significant association between healthcare utilisation and level of burnout at baseline, with a correlation coefficient of -0.33. Some other variables had a rather high correlation coefficient at baseline but were not statistically significant (Table 4). During the MMR programme, there were statistically significant associations between high healthcare utilisation and poor health-related quality of life, high sick leave reported, and poor work ability respectively. During the follow-up, there were statistically significant associations between healthcare utilisation and all variables, with a poorer outcome for the survey variable linked to a higher healthcare use in all cases.

**Table 4:** Associations between health care utilization and survey measure

**Measure**	Pre-MMR		MMR period		Follow-up period	
	Correlation	p	Correlation	p	Correlation	p
Health related quality of life	0.200	0.20	-0.354	0.02	-0.499	0.03
Sick leave	0.016	0.92	0.389	0.01	0.345	0.02
Work ability	-0.278	0.07	-0.354	0.02	-0.701	<0.01
Burnout	-0.335	0.03	0.148	0.34	0.445	<0.01
Anxiety	-0.186	0.23	0.026	0.87	0.367	0.02
Depression	-0.217	0.16	0.071	0.65	0.501	<0.01

Results are reported as the correlation between survey responses and health care utilization, including visits at stress rehabilitation clinic during all measured time points. The matching of time points is as illustrated in Fig. 1. Higher level of health-related quality of life and work ability means better health while for other variables a higher level means worse health

## Discussion

This longitudinal study follows a carefully monitored patient group with established stress-induced exhaustion disorder, which underwent a 24-week MMR programme at a Stress Rehabilitation Clinic. We found that healthcare utilisation remained high during the whole study period. The healthcare utilisation reduced somewhat during the study period, though not statistically significantly. Thus, the healthcare utilisation did not decrease in the way we expected in our preliminary hypothesis. At the end of the study, there were still patients with stress-induced exhaustion disorder and residual health problems that needed further clinical support.

Even though our study lacked signs of a distinct decrease in healthcare consumption after MMR, there were improvements for most of the health parameters during the study period. In most cases the improvements could be considered clinically important, for instance regarding anxiety and depression, where few had severe problems after follow-up, which is in line with most previous research [6, 9, 12]. Still, some residual health problems remained for a considerable number of the patients during the study time. This was shown by reduced health-related quality of life, a high level of clinical burnout and sick leave, and low work ability at the end of the study, despite all of them showing both clinically and statistically significant improvements between pre-MMR and MMR as well as between MMR and end of follow-up. For health-related quality of life, there was an improvement in both the mean and median score from 0.59 to 0.70 at end of follow-up. Still, at the end of the study it remained low in comparison with what has been shown in previous Swedish general population studies, where mean scores for health-related quality of life were around 0.80 [28].

Healthcare consumption was correlated with persistent health problems at the end of follow-up, which can be interpreted as the patients’ remaining health problems still needing care and attention. For the same relationships before and during MMR, there were few statistically significant associations. Most notably, there was a high level of burnout associated with less healthcare utilisation before MMR. This finding, which is not in line with our expectation, might indicate that patients who had delayed contact with healthcare had increased their level of burnout. It could also be a spurious association as a consequence of our small sample, and additionally to this the many explorative analyses conducted in our study especially in regard to associations between health care consumption and survey variables. Further studies are needed to get a better understanding of the patterns observed by us.

Our study was restricted to a short follow-up period. It would be valuable to follow-up our patients over a longer time period as it has been seen in a Swedish study that, at 7 years follow-up, as many as a third of the patients with stress-induced exhaustion disorder still fulfilled the criteria for exhaustion syndrome [6]. Furthermore, it would also have been valuable to evaluate other health problems, for instance infections, which a previous Swedish study reported to be common two years before the diagnosis of stress-induced exhaustion disorder [19].

We have not been able to identify similar longitudinal studies that have examined healthcare utilisation in patients with burnout or stress-induced exhaustion disorder. Our main interest was not in the health care consumption during MMR treatment as this is supposed to cover most of the health care consumption related to the patients’ disease. It is though interesting to note that we had on average 32 visits per patient at the Rehabilitation clinic during the MMR. This could be interpreted as many as the MMR program itself consists of 22 weekly three-hour group sessions. In another Swedish study conducted by Clason van de Leur et al [12], there were even more visits, with around 40 visits planned for the patient during their 24 week long MMR programme. Their study reported a higher level of anxiety and depression before MMR, while exhaustion was on a similar level. Their improvements were similar to those in our study and thus it might not help to increase the number of meetings for patients, while other solutions might be needed instead to improve the rehabilitation process.

In our study, we describe the rehabilitation process and how patients improved by attending MMR. An obvious weakness of our study is that we lack a reference group and therefore cannot conclude that changes were linked to the MMR, such as the level of healthcare consumption. Still, our study will contribute with important additional information to both researchers and policy makers as the documentation of healthcare utilisation and health-related quality of life for patients with stress-induced exhaustion is very limited. Even if the availability of data within the Swedish system is high, we were still unable to obtain all the valuable information related to healthcare consumption, for instance information about all the utilisation of occupational healthcare, which is important for the treatment of these patients but likely limited during MMR. Our judgement is, however, that such information is likely to only marginally affect our results, and that our results should still be valid.

In our study, relatively few patients were eventually recruited, and there may be some selection bias, which limits generalisability from our study to patients in general with stress-induced exhaustion disorder. It is also well known that patients that are referred to the Stress Rehabilitation Clinic often have more severe stress-related exhaustion disorders in comparison with all such patients seeking care at the primary care units. The study was not dimensioned for our the main aim of current study but instead to detect a decrease of 25% in sick leave level, and the 36 participants needed for this purpose was reached. Thus, the study population can still be considered as sufficiently large. However, we considered our study sample to be too small to study the significance of sex, age, education and type of working organisation on recovery from stress-induced exhaustion disorder and healthcare utilisation. It would be valuable with larger future studies for a better understanding of how these characteristics interact with the recovery from stress-induced exhaustion disorder. Despite having a sufficiently dimensioned study for the purpose of the data collection, the many analyses conducted in our study limits the interpretation of results and caution should be taken as spurious associations might have appeared. Some of the results should then rather be seen as exploratory and descriptive analyses, for instance the correlation analyses, which we have also informed about in the method section. However, in regard to the correlations, most of these shown very strong patterns and it is therefore likely that our results would be repeated also for larger samples.

For some of the aspects that we cover in our study, such as return to work and the level of burnout, depression and anxiety, a considerable amount has already been published, while there is only limited amount of data about healthcare utilisation and health-related quality of life, which indicates a need for further studies to complement our contribution. There are still knowledge gaps for the effectiveness of MMR and we were therefore unable to evaluate the cost-effectiveness of the MMR conducted for our patients. A future comparative study for this would be needed to combine with our data.

## Conclusions

In our study, we show that healthcare consumption remains on a high level for patients with stress-induced exhaustion disorder that take part in MMR at a Stress Clinic one year after the end of MMR. Furthermore, the patients report on average a reduced health-related quality of life at 1-year follow-up despite the treatment for stress-induced exhaustion. Patients with poor health results were also those who had the highest healthcare consumption. Further studies are needed to evaluate the effectiveness of MMR and to understand for whom it might be effective, as well as cost-effective.

## Data Availability

The datasets generated and/or analysed during the current study are not publicly available because the Swedish Data Protection Act (1998:204) does not permit sensitive data on humans to be freely shared. The datasets are available based on ethical permission from the Regional Ethical board in Umeå, Sweden, from one of the authors (Therese Eskilsson).

## Abbreviations

HAD: The Hospital Anxiety and Depression Scale
ICD-10: 10th revision of the International Statistical Classification of Diseases and Related Health Problems
MMR: Multimodal rehabilitation
SF-36: Short Form 36
SMBQ: The Shirom Melamed Burnout Questionnaire
WAI: Work Ability Index
